# MicroRNA-150 enhances radiosensitivity by inhibiting the AKT pathway in NK/T cell lymphoma

**DOI:** 10.1186/s13046-017-0639-5

**Published:** 2018-01-31

**Authors:** Shao Jie Wu, Jun Chen, BingYi Wu, Yu Jue Wang, Kun Yuan Guo

**Affiliations:** 1 Department of Hematology, Southern Medical University, Zhujiang Hospital, 253# industry road, Guangzhou, Guangdong 510282 China; 2 Department of Radiotherapy, Southern Medical University, Zhujiang Hospital, 253# industry road, Guangzhou, Guangdong 510282 China; 3 Department of Laboratory Animal Center, Southern Medical University, 1838 Guangzhou North Road, Guangzhou, Guangdong 510515 China

**Keywords:** MicroRNA-150(miR-150), NK/T cell lymphoma, PI3K/AKT/mTOR pathway, Radiosensitivity, Apoptosis

## Abstract

**Background:**

Radioresistance is a major challenge during the treatment of NK/T cell lymphoma. This study aimed to investigate the potential role of MicroRNA-150 (miR-150) in increase the sensitivities of NK/T cell lymphoma to ionizing radiation.

**Results:**

In this study, we found that miR-150 was significantly decreased in NK/T cell lymphoma tissues and cell lines. Low expression of miR-150 was positively associated with therapeutic resistance in 36 NK/T cell lymphoma cases. Our further in vitro and in vivo studies illustrated that overexpression of miR-150 substantially enhanced the sensitivity of NK/T cell lymphoma cells to ionizing radiation treatment. Furthermore, luciferase reporter assays in NK/T cell lymphoma cells transfected with the AKT2 or AKT3 three prime untranslated region reporter constructs established AKT2 and AKT3 as direct targets of miR-150. The phosphatidylinositol 3-kinase inhibitor LY294002 was used to inhibit Akt to verify miR-150 increase NK/T cell lymphoma cell radiorsensitivity through suppress the PI3K/AKT/mTOR pathway.

**Conclusions:**

Taken together, this study demonstrates that miR-150 might serve as a potential therapeutic sensitizer through inhibition of the AKT pathway in NK/T cell lymphoma treatment.

**Electronic supplementary material:**

The online version of this article (10.1186/s13046-017-0639-5) contains supplementary material, which is available to authorized users.

## Background

NK/T cell lymphoma (NKTL) is diagnosed more commonly in Asia and Latin America than in Western countries and accounts for 5–10% of all malignant lymphomas in China [1, 2]. NKTL is a highly invasive tumor with a short doubling time and poor prognosis. Radiotherapy is usually regarded as the main treatment, however, the cellular response to irradiation is complex. The treatment effects also depend on many factors. For example, the dose, dose rate, and fractionation play an equally important role in deciding the fate of the cell. One of the main causes of failure in radiotherapy is radioresistance [3–5]. Therefore, to develop effective radiotherapy strategies in the future, the knowledge of how radioresistance develops at the molecular level is needed.

MicroRNAs (miRNAs) are a class of short, highly conserved, noncoding RNAs that function as negative post transcriptional regulators of the target genes [6]. Recently, growing evidence has shown that miRNAs are aberrantly expressed in the development and progression of variety of human cancers [7]. Increasing evidence has confirmed that miRNAs can modulate the radiosensitivity of cancer cells, suggesting the potential to improve the efficacy of radiotherapy [8, 9]. miR-150 has recently been identified as a key regulator of immune cell differentiation and activation, as it is preferentially expressed in mature B and T cells as well as NK cells and other cell types of the hematopoietic system [10–12]. MicroRNA-150 (miR-150) has been validated as an oncogene or tumor suppressor in several human malignancies [13, 14]. Previous reports have documented that miR-150 was frequently downregulated in NKTL [15]. However, the role and mechanisms of miR-150 in NKTL therapeutic effects remain elusive. This study aimed to investigate the potential role of miR-150 in increasing the sensitivities to ionizing radiation and through suppress the PI3K/AKT/mTOR pathway of NKTL.

## Methods

### Cell lines and cell cultures

We used 5 cell lines as NK/T-cell lymphoma leukemia cell lines, which are commonly showing CD2^+^, sCD3^−^, CD3ε^+^, CD5^−^, CD56^+^, TCRαβ^−^ and TCRγδ^−^ phenotypes, including KHYG-1, NK-92, HANK-1, SNK-1, SNK-6. Of these, HANK-1, SNK-1 and SNK-6 are EBV^+^. Those cell lines were maintained in alpha-MEM (Minimum Essential Medium) supplemented with 10% fetal bovine serum (Invitrogen, Carlsbad, CA) and 100 U/ml recombinant IL-2. NK cells showing the sCD3^−^ and CD56^+^ phenotype were collected from 6 healthy donors as a control using a magnetic cell sorting system and flow cytometry (Additional file 1: Figure S1). All cell lines were cultured at 37 °C in a humidified atmosphere of 5% CO_2._

### Patients and therapy response evaluation

Paraffin-embedded tissues of 36 primary NKTL were collected from 22 nasal, 6 pharyngeal, 5 gastrointestinal tract and 3 lung, and obtained from the Department of Pathology of Zhujiang Hospital, Southern Medical University, between January 2010 and January 2016. These were assessed according to Ann Arbor Staging [16]. None of the patients received radiotherapy before biopsy sampling. The response of radiotherapy was assessed clinically for primary lesion based on CT (computed tomography) and PET (positron emission tomography)/CT 1 month after treatment according to the following criteria: complete response (CR) was defined as the complete disappearance of all assessable lesions, partial response (PR) was defined as a reduction of the sum of the lesions by 50% or more and no progression of assessable lesions, stable disease (SD) was indicated by a reduction of < 50% or an increase of < 25% in tumor size. All these conditions were required to last for at least 4 weeks with no appearance of new lesions. Progressive disease (PD) was defined as an increase of 25% in tumor size or the appearance of new lesions. The PR, SD and PD define as Non-CR.

### Quantificational real-time polymerase chain reaction (qRT-PCR) for quantitative analysis of miR-150

Total RNA was extracted from the cells and tissues using TRIzol reagent according to the manufacturer’s protocol. The reverse transcription and polymerase chain reaction (PCR) primers for miR-150 and U6 were obtained from RiBoBio. Reverse transcription primersfor miR-150 were 5′-CTCAACTGGTGTCGTGGAGTCGGCAATTCAGTTGAGCACTGGTA-3′. The PCR primers for miR-150 were 5′-ACACTCCAGCTGGGTCTCCCAACCCTTGTACCA-3′ and 5′-CTCAACTGGTGTCGTGGA-3′. The PCR primers for U6 were 5′-CTCGCTTCGGCAGCACA-3′ and 5′-AACGCTTCACGAATTTGCGT-3′. The cDNA library was synthesized using the PrimeScript RT reagent kit. For quantification of mature miRNA, cDNA was generated using specific stem-loop universal primers. qRT-PCR for miRNA and mRNA was performed using SYBR Premix Ex Taq II and was measured using an ABI 7500 Sequence Detection System. U6 was used as the internal control.

### Plasmid construction, lentivirus production and transduction

The hsa-miR-150-5p gene fragment was synthesized chemically and cloned into lentiviral vector pLVX-ZsGreen-miRNA-Puro. The constructed recombinant plasmid pLVX-ZsGreen-miR-150-5p-Puro/pLVX-ZsGreen-miRNA-Puro, pspax2 and pMD2G was transfected to packaging cell line 293 T, and the packaged virus was determined for titer. Recombinant lentivirus particles harvested and concentrated, then 1 × 10^4^/L NK-92/Hank-1 cells were seeded into 24 well plates, we added the virus suspension into plates by Polybrene concentration gradient dilution method, the infection rate was low (about 30–40%) after 72 h by fluorescence microscope, then GFP-positive cells were selected transfer to 96 well plates for further culture by flow cytometry cell sorting, GFP-positive clones were expanded to construct stable cell line, and tested for expression level of miR-150 by qRT-PCR. The experiment was divided into three groups, including mock group, miR-control group, and miR-150 group.

### Cell proliferation assay

1 × 10^6^ NK-92 or Hank-1cells of above three groups were irradiated for each well with 0, 2, 4, 6, 8, and 10 Gy, respectively. Cells were subjected to radiation exposure by a 6-Mv X ray (isocentric) linear accelerator. The dose rate was fixed as 300 cGy/min for three groups. The SSD (source to surface distance) was a 100 cm radiation field for 10 × 10 cm, Three days later, cell viability was measured by MTT assay. At least 3 independent experiments were performed.

### Clonogenic assay

1 × 10^6^ NK-92 or Hank-1cells were treated with 0, 2, 4, 6, 8, and 10 Gy of irradiation, respectively. Twelve days later, the cells were fixed with methanol and stained using 1% crystal violet in 70% ethanol. The colonies containing 50 or more cells were counted according to our previous study [17] . The survival curve was derived from a multi-target single-hit model, y = 1–1-exp.(−D/D0)n [18]. The radiation sensitivity enhancement ratio (SER) was measured according to the multi-target single-hit model. In some experiments, cells were treated with LY294002 starting 1 h before radiation for a duration of 24 h. At least 3 independent experiments were performed.

### Flow cytometry analysis of cell cycle and apoptosis

1 × 10^6^ NK-92 or Hank-1 cells of above miR-control and miR-150 group for each well were irradiated with 6 Gy, the cells were collected at 48 h after radiation. Annexin V fluorescein isothiocyanate and propidium iodide stains were used to determine the percentage of cells undergoing apoptosis. The apoptosis assay was conducted using the protocol supplied by the manufacturer. Each sample was then subjected to analyses by flow cytometry. At least 3 independent experiments were performed.

### Luciferase reporter assay

The 3′ untranslated region (UTR) of AKT2 and AKT3 containing the putative miR-150-binding site was amplified from genomic DNA via PCR using the following primers: wt-AKT2 F, 5′-GCAGTCTGCCCACGCAGAGG-3′, and Rv, 5′-TACAG ATGGATAGCTAGTTTA-3′, wt-AKT3 F, 5′-GTCTCTTTCATTCTGC TACTTCAC -3′, and Rv, 5′-GTAAAATGCCCTTTAACCCCCGT-3′. Mutation in 3′-UTR of both genes with miR-150 target binding site deleted was generated with the Quick Change Site-Directed Mutagenesis kit. Both the wild and mutant types of AKT2 and AKT3 genes were cloned into the psiCHECK-2 vector immediately downstream of the Renilla luciferase gene. A luciferase reporter construct containing the miR-150 consensus target sequence served as the positive control, and the psiCHECK-2 vector was used as the internal control. Cells were co-transfected with psiCHECK-2 firefly luciferase reporter (50 ng), psiCHECK-2 Renilla luciferase reporter (10 ng), and miR-150 (50 nM) or miR-control (50 nM) with Lipofectamine 2000 reagent. Cell lysates were prepared using passive lysis buffer for 48 h upon transfection, and luciferase activity was measured using the Dual-Luciferase Assay kit and normalized to firefly luciferase activity. Three independent experiments were performed in triplicate.

### Western blot (WB)

Cells were collected in ice-cold PBS 48 h after transfection and lysed on ice in cold modified RIPA buffer supplemented with protease inhibitors. The concentration of protein was determined by the BCA Protein Assay Kit. Then equal amount of protein were analyzed by SDS-PAGE. Gels were electroblotted onto nitrocellulose membranes, blocked in PBS/dry milk/0.05% Tween-20, membranes were incubated at 4 °C over night with first antibodies (Monoclonal rabbit anti-human anti-Akt (ab126433), anti-p-Akt (ab8805), anti-caspase 3 (ab136812), anti-AKT2 (ab38513), anti-AKT3 (ab152157), anti-mTOR (ab2732) and anti-p-mTOR (ab84400) were all purchased from Cell Signaling Technology (Beverly, MA, USA), and monoclonal mouse anti-human anti-Alpha-Tubulin (ab7750) and anti-GAPDH (ab8245) were purchased from Kang Chen Bio-tech (shanghai, china), according to the manufacturer’s instructions.

### In vivo experiment

Four-week-old male severe combined immunodeficient mice were injected subcutaneously with 2 × 10^5^ Hank-1-miR-150 or Hank-1-miR-control cells, separately. When the tumor mass became palpable (about 110 mm^3^), mice in each group were randomly divided into subgroups, including control group, and IR-treated group. Each group contained six mice, and there was no difference in tumor size among the groups. Using this model, we monitored the efficacy of IR (2 Gy/2 days, total 40 Gy) in the miR-150 and miR-control groups, respectively. For the control group, dimethyl sulfoxide alone (100% DMSO, 40 uL, intraperitoneal injection every 2 days) was delivered to the mice. Tumor volume (V) was monitored by measuring the length (L) and width (W) of the tumor with calipers and was calculated with the formula V = (L*W^2^) *0.5. All the procedures were in accordance with the guidelines of the Animal Ethics Committee of Southern Medical University.

### Statistical analysis

Statistical analysis was performed with SPSS 16.0 software. All data are presented as the mean ± SD, and analyzed using Student’s t-test or analysis of variance (ANOVA) for normal distribution. Comparisons between two/three groups were performed with the Mann–Whitney U-test or the Kruskal-Wallis test for non-normal distribution. A Chi-square test was used to analyze the relationship between miR-150 and the clinical characteristics. *P*-values of < 0.05 were considered statistically significant.

## Results

### Low levels of miR-150 were frequently found in NKTL tissues and cell lines

**Fig. 1:**
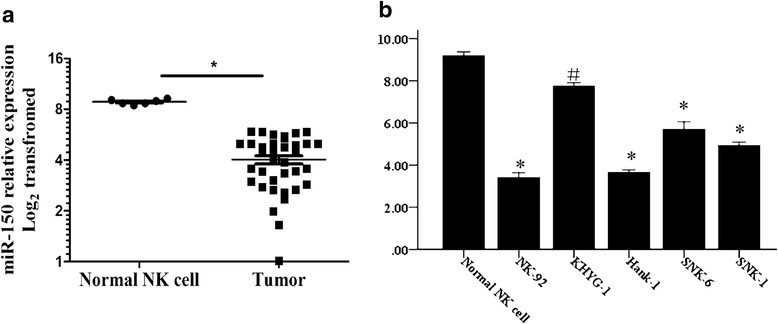
The expression levels of miR-150 in NKTL tissues and cell lines examined by qRT-PCR. **a** Expression of miR-150 in 36 samples of NKTL and normal NK cell (Student’s t-test); **b** miR-150 expression levels varied in different NKTL cell lines (Post Hoc test of ANOVA). ^#^*P* < 0.05, ^*^P < 0.01

In this study, we detected miR-150 expression levels in NKTL tissues from 36 patients. The expression levels of miR-150 were obviously lower in the tumor tissues than those in normal sCD3^−^ and CD56^+^ NK cells (9.10 ± 0.19 vs 4.01 ± 0.22, *P* < 0.05). In addition, among five NKTL cell lines examined, four of them (NK-92, SNK-6, HANK-1, SNK-1) had significantly lower expression levels of miR-150 than NK cells obtained from normal peripheral blood. (*P* < 0.05, Fig. 1).

### miR-150 expression was positively correlated with the treatment response of NKTL patients

As shown in Table 1, low expression of miR-150 was closely correlated with the viral load of Epstein-Barr virus (EBV) (EBV ≧ 50 copy/ml severse as positive) (*P* = 0.007). No significant association was found between miR-150 expression and other clinico-pathological features, such as the patient’s gender, age, clinical stage, and IPI stage (*P* > 0.05). In our enrolled cases, CR, PR, NC, and PD were achieved in 12, 4, 5, and 15 patients at the evaluation time, respectively. Moreover, miR-150 expression was also the factor that showed a positive correlation with treatment response in our enrolled cases, in which low expression of miR-150 was observed more frequently in patients from the non-CR subset than those in the CR subset (*P* = 0.000).

**Table 1:** Correlation between the expression of miR-150 and clinicopathologic features in NKTL

		miR-150 expression level		
	All cases	Low expression	High expression	*P* value
Gender				0.278
female	16	12 (75%)	4 (25%)	
male	20	12 (55%)	8 (40%)	
Age				0.637
≥ 40 years	15	10 (66.7%)	5 (33.3%)	
< 40 years	21	14 (77.8%)	7 (22.2%)	
Therapy response				0.000
CR	12	3 (25%)	9 (75%)	
Non-CR	24	21 (87.5%)	3 (12.5%)	
Clinical stage				0.133
I	3	1 (33.3%)	2 (66.7%)	
II	7	3 (42.8%)	4 (57.2%)	
III	18	15 (83.4%)	3 (16.6%)	
IV	8	5 (62.5%)	3 (37.5%)	
IPI stage				0.853
1	18	13 (72.2%)	5 (27.7%)	
2	14	9 (64.2%)	5 (35.7%)	
3	2	1 (50%)	1 (50%)	
4	2	1 (50%)	1 (50%)	
EBV				0.007
normal	10	3 (30%)	7 (70%)	
increase	26	21 (80.7%)	5 (19.2%)	

Mean age; *WHO* World Health Organization, *CR* complete response, Non-CR (including *PR* partial response, *NC* no change, *PD* progressive disease)

### miR-150 enhanced the sensitivity of NKTL cells to IR

**Fig. 2:**
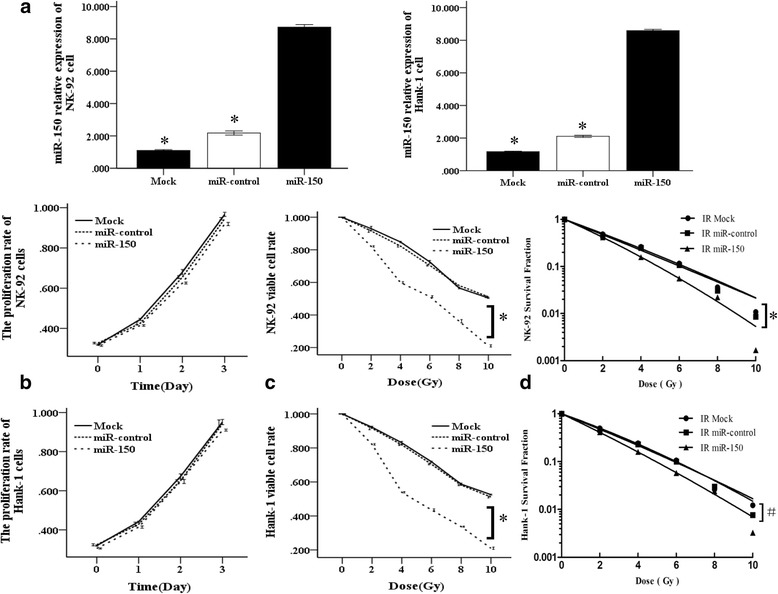
miR-150 enhances the sensitivities of NKTL cells to IR in vitro. **a** qRT-PCR analysis of miR-150 in NK-92 and Hank-1 NKTL cells after overexpression of miR-150 (*P < 0.01 vs miR-150 group ) (Student’s t-test). **b** MTT assay showed overexpression of miR-150 in NK-92 and Hank-1 cells had no impact on the proliferative activity. Data are expressed as absorbance (Post Hoc test of ANOVA). **c** The percentages of viable cells of NK-92 and Hank-1 cells transfected with miR-150 compared with negative control, which were exposed to IR as indicated for 72 h by MTT assay (*P < 0.01 vs control group after 10 Gy radiation) (Post Hoc test of ANOVA). **d** miR-150 enhanced the growth inhibition of NKTL cells exposed to IR in vitro. miR-150 transfected and miR-control cells were treated with IR at the indicated doses of IR for 15 days. The colonies were stained and counted, and survival curves were constructed from three independent experiments (Kruskal-Wallis test). (^#^*P* < 0.05 / ^*^P < 0.01 vs control group after 10 Gy radiation)

To explore the underlying mechanism involved in the role of miR-150 in the treatment response of NKTL cells, we induced miR-150 expression upon lentiviral infection in NK-92 and Hank-1 cells, which had the lowest levels of miR-150 among our available NKTL cell lines. Increased expression of miR-150 was confirmed by qRT-PCR (Fig. 2a). In the MTT cell proliferation assay, there were no difference between three groups from two cell lines (*P* > 0.05). we tested whether miR-150 increased NKTL cell sensitivity to radiotherapy in vitro. NK-92 and Hank-1 cells were treated with various clinical doses of IR. As shown in Fig. 2c, IR was readily cytotoxic to NK-92 and Hank-1 cells. However, IR resulted in a higher level of cell death in miR-150 group compared to the miR-control and mock group (*P* < 0.05), however, there were no differences between the miR-control and mock group (*P* > 0.05). In addition, we applied colony formation assay by GraphPad Prism 5 software to further confirm that miR-150 increased the sensitivity of NKTL cells to IR in vitro. NKTL cells formed fewer colonies in miR-150 groups compared to other group (Fig. 2d), according to the linear quadratic model-fitted cell survival curve. The corresponding biological parameters of the survival curve are shown in Table 2. SERD0, SERDq, and SERSF_2_ were 1.36, 2.050, and 1.064, respectively. These results provided evidence that miR-150 could increase the sensitivity of NKTL cells to radiotherapy in vitro.

**Table 2:** Radiation biology parameters by single-hit multi-target model

Groups	D0	Dq	SF_2_	SER_D0_	SER_Dq_	SERs_F2_
Control	2.7	0.37	0.474	–	–	–
miR-150	1.98	0.18	0.417	1.363	2.050	1.064

### miR-150 promoted IR-induced apoptosis in two cell lines in vitro

**Fig. 3:**
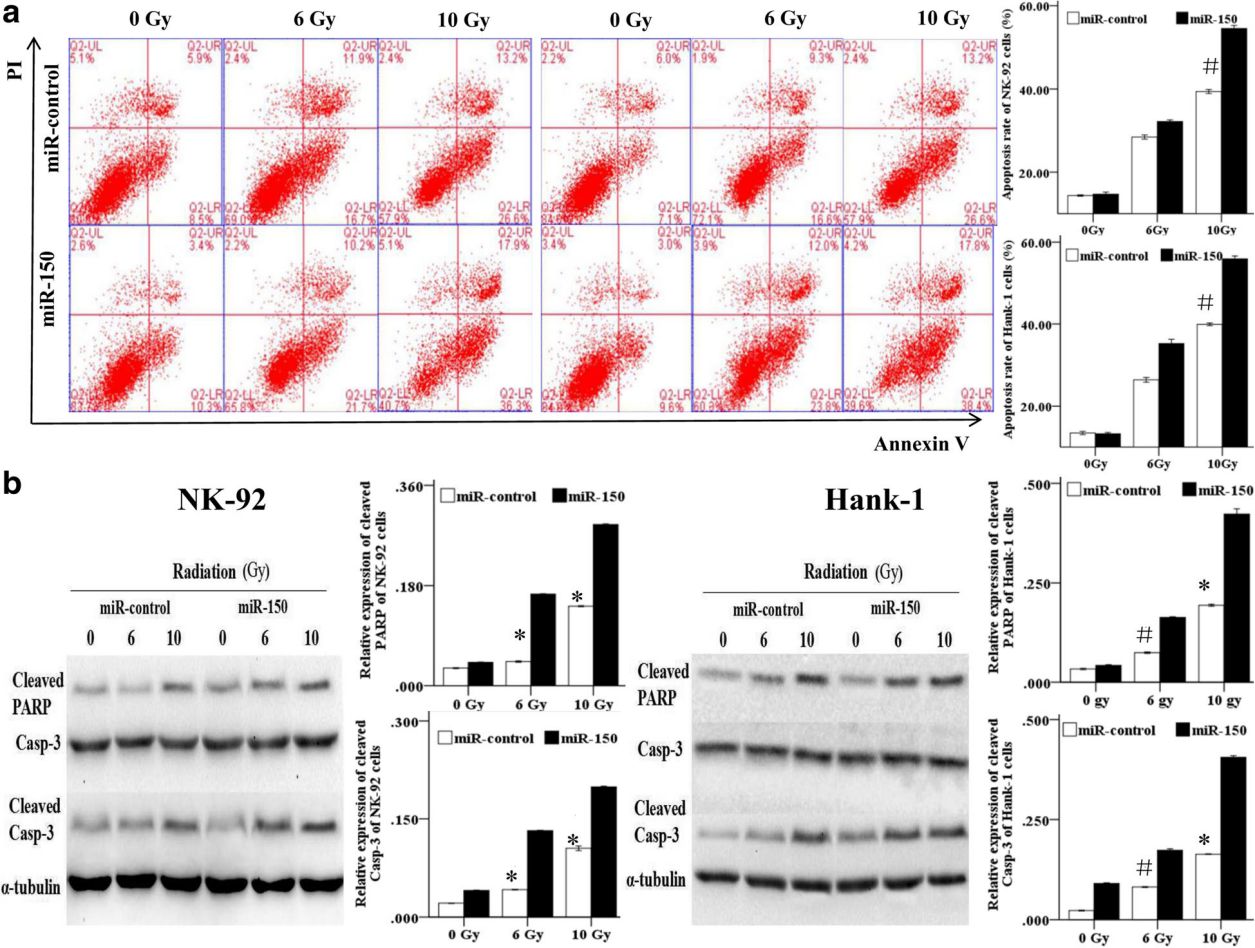
miR-150 promotes the apoptosis of Hank-1 and NK-92 cells induced by IR in vitro. **a** miR-150 transfected and miR-control Nk-92 and Hank-1 cells were treated with IR (6 Gy and 10 Gy). The cells were collected and stained with PI and Annexin V (Mann-Whitney U-test). **b** After treatment with IR, the cleavages of caspase 3 and PARP was detected by Western blot analysis. ^#^*P* < 0.05, ^*^P < 0.01

To test whether the miR-150-induced sensitivity to IR was due to activation of apoptosis, we examined the effects of miR-150 on apoptosis in NKTL cell lines using flow cytometry. As shown in Fig. 3a, overexpressions of miR-150 significantly promoted the radiation-induced apoptosis of NK-92 cells [(39.8 ± 1.04)% vs (54.2 ± 1.12)%, *P* < 0.05] and Hank-1 cells [(39.8 ± 1.24)% vs (56.2 ± 1.04)%, *P* < 0.05] for Annexin V/PI analysis when treated with IR (10 Gy). As illustrated in Fig. 3b, the levels of cleaved caspase-3 [(0.16 ± 0.02 vs 0.40 ± 0.01,*P* < 0.05)] and cleaved PARP [(0.19 ± 0.03 vs 0.42 ± 0.01, *P* < 0.05)] in Hank-1 cells and the cleaved caspase-3 [(0.10 ± 0.02 vs 0.19 ± 0.01,*P* < 0.05)] and cleaved PARP [(0.14 ± 0.03 vs 0.28 ± 0.01, *P* < 0.05)] in NK-92 cells were increased in miR-150 group compared to the miR-control group after treated with IR (10 Gy) by western blot analysis. These data revealed that miR-150 promotes IR-induced apoptosis in NKTL cells.

### Both AKT2 and AKT3 are direct targets ofmiR-150 in NKTL cells

**Fig. 4:**
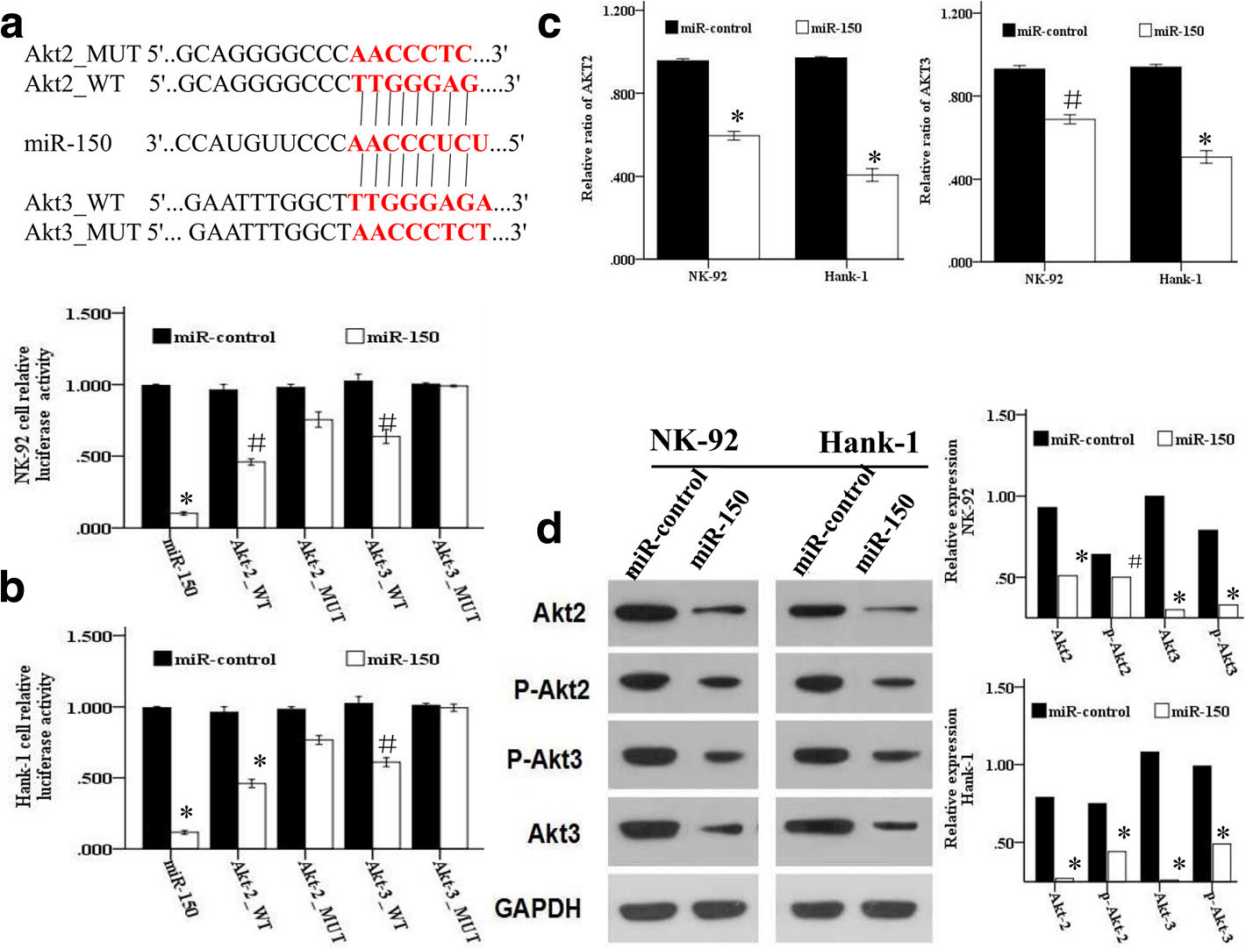
AKT2 and AKT3 are direct targets of miR-150 in NKTL. **a** Schematic representation of AKT2 and AKT3 UTR showing putative miR-150 target sites; **b** Relative luciferase activity were performed to detect the expression of wild-type/mutant AKT2 and AKT3 upon transfection with miR-150 or miR-control (normalized to firefly luciferase activity); **c** Relative ratio of AKT2 and AKT3 reporter constructs in Hank-1 and NK-92 cell lines, co-transfected with miR-150 or miR-control is shown; **d** Western blot analysis showed the expression levels of total AKT, p-AKT, AKT2 and AKT3 proteins in NKTL cells treated with miR-150 and miR-control. (Student’s t-test). ^#^*P* < 0.05, ^*^P < 0.01

We searched for miR-150 targets using three publicly available databases (TargetScan, Pictar and miRBase). We then used WB analysis to assess expression in various candidate targets, including c-Myb, FOXP1, AKT2, AKT3, BCAP, PRL1, c-Raf, MSK1, MIB1 and E2F3 in NK-92 and Hank-1 cells transduced with miR-150 or empty vector. The PI3K signaling pathway is usually activated in lymphoma, and inactivation of this pathway impairs DNA repair following radiation, then, we found that levels of both AKT2 and AKT3 were reduced in all miR-150 transductants (Fig. 4d). To demonstrate the direct inhibition by miR-150, luciferase reporter containing wild-type AKT2 and AKT3 3’UTR sequences or their mutant-derivates with deletion of putative miR-150-binding sites were cotransfected into both NKTL cell lines with miR-150 (Fig. 4a). As shown in Fig. 4b, miR-150 suppressed luciferase activities depending on the presence of miR-150 binding in the wild-type AKT2 and AKT3 3’UTR. Upon 3’UTR mutation of AKT2 and AKT3, miR-150 can not bind to its target site, resulting in increased luciferase activity. The ratio of wild-type AKT2 and AKT3 were decreased upon miR-150 overexpression in Hank-1 and NK-92 cell lines (Fig. 4c). Morever, overexpression of miR-150 significantly suppressed the protein expression of AKT2 and AKT3 in NK-92 and Hank-1 cells using WB analysis (Fig. 4d), All these results together suggested that AKT2 and AKT3 direct targets of miR-150 in NKTL cells.

### miR-150 induces NKTL cells radiosensitivity by inhibiting the PI3K/AKT/mTOR pathway

**Fig. 5:**
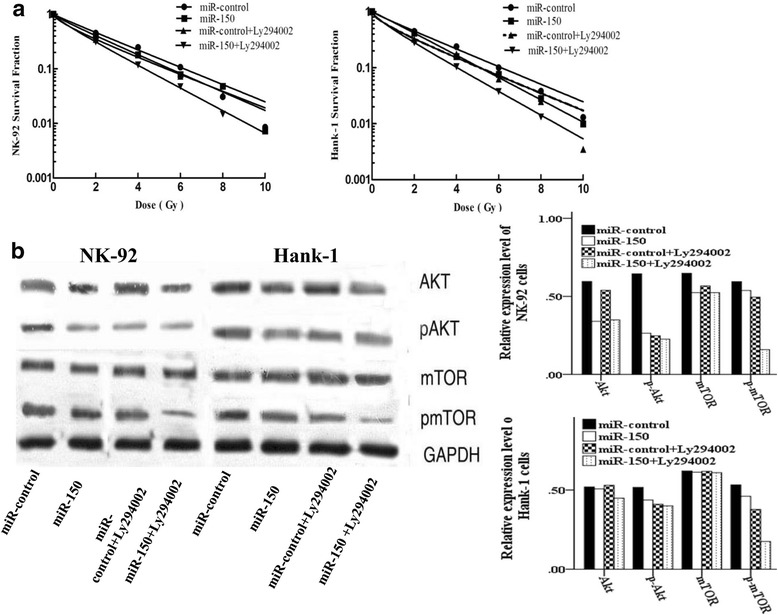
The PI3K tyrosine kinase inhibitor LY294002 synergistic effect of miR-150. **a** The NKTL cells lines NK-92 and Hank-1 were transfected with miR-150 or miR-control. The cells were then treated with or without LY294002 for 1 h before exposure to different doses radiation. Survival fractions were calculated and analyzed (Kruskal-Wallis test); **b** Western blot analysis of p-mTOR and p-Akt levels in NK-92 and Hank-1 cells transfected with miR-150 or miR-control, then treated with LY294002 for 1 h, and finally exposed to a 6 Gy dose of radiation for another 24 h. ^#^*P* < 0.05, ^*^P < 0.01

mTOR is the downstream molecular of AKT signaling pathways. We found that overexpression of miR-150 reduced the expression of AKT meanwhile decreased the expression of p-mTOR by WB analysis. Moreover, we found that exposure to the tyrosinekinase inhibitor LY294002 activating the effect of miR-150 resulted in decrease of p-AKT/p-mTOR expresssion and formed colonies after different dose of radiation. There were statistical difference between the four groups in NK-92 cell (Χ^2^ = 11.26 *P* = 0.021) and Hank-1 cell (Χ^2^ = 10.38 *P* = 0.016) after 10 Gy radiation, and the total clone formation rate was minimum in the miR-150 + LY294002 group according to mean rank (Fig. 5a and b). Those function experiment confirmed that AKT pathway was the mechanism by which miR-150 sensitizes cells toward radiotherapy.

### miR-150 enhanced the sensitivity of Hank-1 cells to IR in vivo

**Fig. 6:**
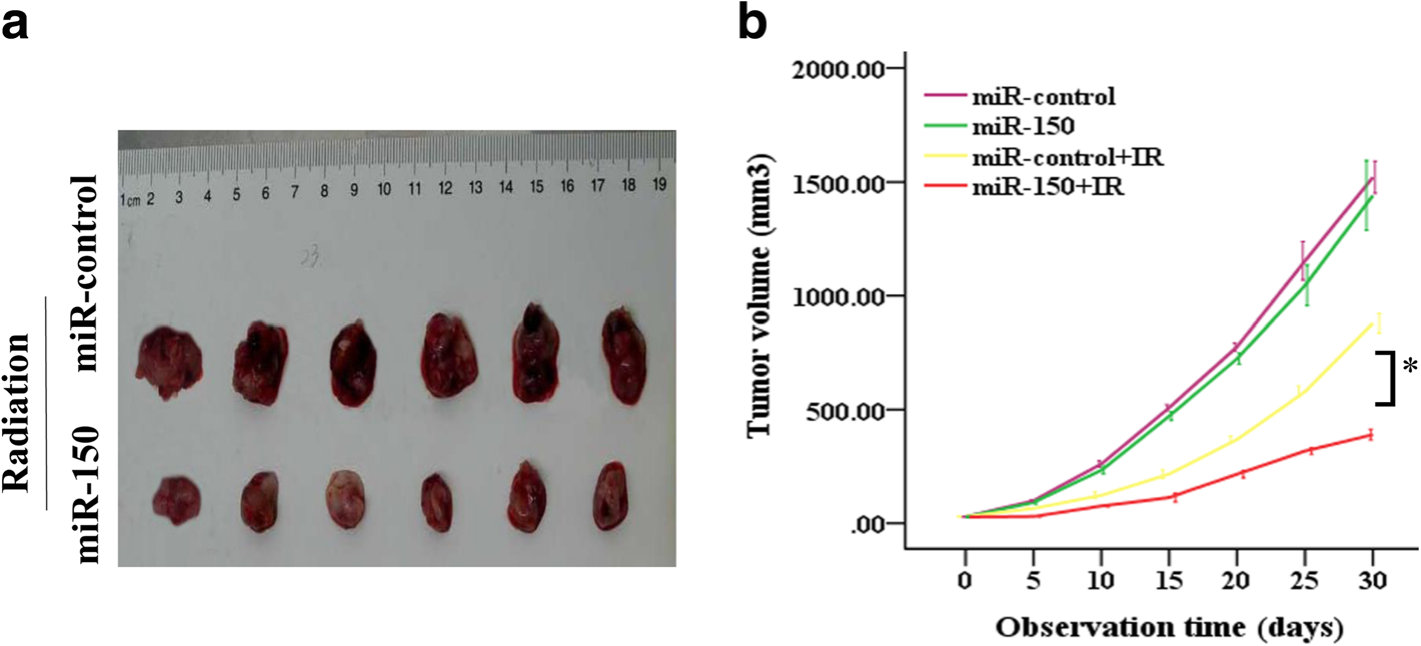
miR-150 enhances the sensitivities of Hank-1 cells to IR in vivo. **a** Representative images of xenograft tumours in NODSCID-12 IL2RG−/− (NSI) mice induced by Hank-1-miR-150 or Hank-1-miR-control cells. The tumor volumes after treatment with IR within 30 days of two groups. **b** The tumor volumes were measured and recorded every 5 days (the groups related to IR). The tumor growth curves were created from 6 mice in each group (Post Hoc test of ANOVA). ^*^P < 0.01

Freshly isolated Hank-1-miR-control and Hank-1-miR-150 cells were transplanted into NODSCID-12 IL-2 RG−/− (NSI) mice(purchased from Guangzhou Institutes of Biomedicine and Health (GIBH)) to establish P1 NKTL mice according to protocols [19]. We next examined the anti-tumor effect of miR-150 on NKTL xenografts treated with IR. The tumors were irradiated with 2 Gy every 2 day (total dose = 40 Gy) after tumor mass became palpable, followed by detection of NKTL xenograft growth (Fig. 6). It can be seen that the tumor volume was significantly decreased in the radiotherapy group (from day 10 to 30, *P* < 0.01) and combined therapy group (from day 10 to 30, *P* < 0.01) compared with control group. The tumor volume of control group showed no significant change compared with miR-150 group (from day 5 to 20, *P* > 0.05). The average tumor volume reached 1587.96 ± 83.56 mm^3^ in control group on day 30, while only 449.58 ± 29.20 mm^3^ in combined therapy group, 1448.02 ± 96.99 mm3 in miR-150 therapy group, and 1012.95 ± 78.77 mm^3^ in radiotherapy group. In addition, the tumor volume was significantly decreased in mice of combined therapy group compared with miR-150 therapy (from day 10 to 30, *P* < 0.01) or radiotherapy group (from day 15 to 30, *P* < 0.01).

## Discussion

In the current study, we found a significant decrease of miR-150 in NKTL biopsies compared with that in normal NK cells. This observation is consistent with the results of several previous reports [15, 20]. Our further correlation analysis demonstrated that decreased levels of miR-150 were closely associated with certain aggressive features of NKTL, such as EBV viral load, and several studies have confirmed the association of an aggressive lymphoma to EBV [21–23]. Chen et al. found that overexpression of miR-150 could induce EBV-positive Burkitt lymphoma to differentiate into a more mature stage [21]. Moreover, we found that low expression of miR-150 in NKTL was correlated closely with poor response to treatment. Thus, our reports suggested that the examination of miR-150 expression could be used as an effective additional tool to predict the therapeutic response of NKTL.

Based on the above findings, we hypothesize whether reintroduction of miR-150 could rescue the therapeutic sensitivity in NKTL cells. Until now, several microRNAs have been reported to participate in regulating therapeutic efficacy in cancer therapy [24-26]. Our present research demonstrated, for the first time, that miR-150 could substantially sensitize NKTL cells to radiation either in vitro and in vivo. The efficacy of radiation is generally believed to depend on their ability to trigger apoptosis in tumor cells. Thus, we further examined the effect of miR-150 on the IR-induced apoptosis in NK-92 and Hank-1 cells using flow cytometry and WB. We found that miR-150 could enhance apoptotic cell induced by IR. These findings strongly suggest that overexpression of miR-150 into NKTL cells sensitize NKTL cells to IR-induced apoptosis.

EBV plays an important role in the lymphomagenesis of NKTL [27]. Latent membrane protein 1 (LMP1) is a major oncoprotein that is encoded by one of the EBV genes [28]. This protein contributes to the activation of the AKT pathway in EBV-infected lymphomas, which, in turn, affects cell survival, apoptosis, proliferation, and genomic instability via its downstream target proteins to cause cancer [29]. Moreover, Huang et al. confirmed that AKT signaling pathways may contribute to the angiogenesis, proliferation, and survival of NKTL [26]. It has also been demonstrated that the activation of the PI3K/AKT pathway can result in resistance to radiotherapy and chemotherapy [30, 31], and inhibition of this pathway can increase the sensitivity of cancer cells to therapy [32, 33]. Several studies have confirmed that the miR-150 regulation mechanism was related to AKT activation [15, 34–36]. Luciferase assays confirmed that the mRNA and protein levels of both wild-type AKT2 and AKT3 could be consistently decreased in NKTL cells through transfection with miR-150 (Fig. 4), moreover, overexpression of miR-150 inhabited the PI3K/AKT/mTOR signaling molecules, and that the PI3K tyrosine kinase inhibitor LY294002 enhanced the effects of miR-150, indicating that the PI3K/AKT/mTOR signaling pathway is involved in miR-150 mediated radiosensitivity.

## Conclusions

In summary, our report describes the expression pattern of miR-150 in human NKTL, and low expression of miR-150 may be important in the acquisition of poor therapeutic response of the tumor. Furthermore, functional studies of miR-150 suggest a critical role of miR-150 in enhancing the sensitivities of NKTL to radiotherapy by suppressing PI3K/AKT/mTOR signaling.

## Additional file

Additional file 1: Supplementary Materials and Methods. (DOCX 49 kb)
