# Supplementary material for: MicroRNA-150 enhances radiosensitivity by inhibiting the AKT pathway in NK/T cell lymphoma
Source: J Exp Clin Cancer Res. 2018 Jan 31;37:18. doi: 10.1186/s13046-017-0639-5 (PMC5793389; doi:10.1186/s13046-017-0639-5)
Supplement: Supplementary file 1 — Supplementary Materials and Methods. (DOCX 49 kb) [file 13046_2017_639_MOESM1_ESM.docx]

**Before Purification After Purification 15 days after culture**

**CD3-CD56+ cells were detected by flow cytometry**
